# Supplementary figures and images for: Genome-wide protein phylogenies for four African cichlid species
Source: BMC Evol Biol. 2018 Jan 8;18:1. doi: 10.1186/s12862-017-1072-2 (PMC5784529; doi:10.1186/s12862-017-1072-2)

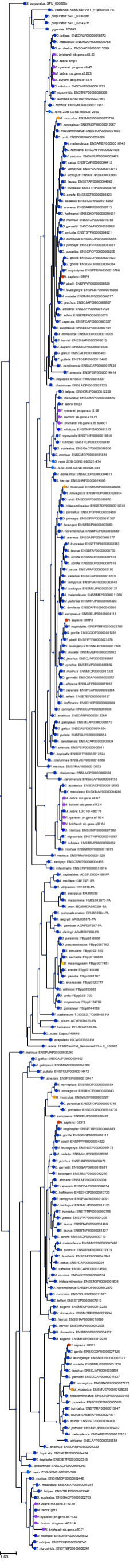

Supplement: Supplementary file 1 — Full tree for the TF351789 family from all 109 species included in the Treefam database. This family contains a number of BMP growth factors belonging to the transforming growth factor beta family. (PDF 3001 kb) [file 12862_2017_1072_MOESM1_ESM.pdf]

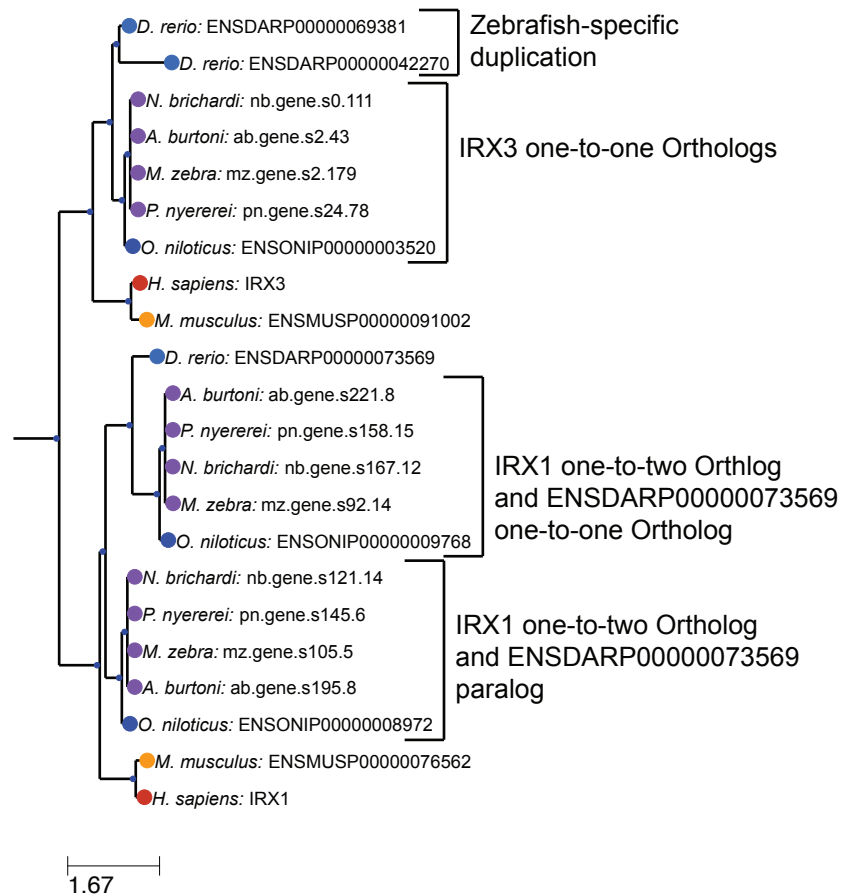

Supplement: Supplementary file 2 — Subtree from the TF319371 family from a limited number of cichlid species and well-studied model organisms. This family contains a number of Iroquois-family of homeodomain transcription factors involved in patterning and other development processes. (PDF 1170 kb) [file 12862_2017_1072_MOESM2_ESM.pdf]
